# Validation of electron-microscopy maps using solution small-angle X-ray scattering

**DOI:** 10.1107/S2059798324005497

**Published:** 2024-06-27

**Authors:** Kristian Lytje, Jan Skov Pedersen

**Affiliations:** a Department of Chemistry and Interdisciplinary Nanoscience Center (iNANO) Aarhus University Gustav Wieds Vej 14 8000Aarhus Denmark; Tsinghua University, People’s Republic of China

**Keywords:** electron microscopy, small-angle X-ray scattering, electron-microscopy validation, structure determination

## Abstract

The determination of the atomic resolution structure of biomacromolecules is essential for understanding details of their function. Traditionally, such a structure determination has been performed with crystallographic or nuclear resonance methods, but during the last decade, cryogenic transmission electron microscopy (cryo-TEM) has become an equally important tool. As the blotting and flash-freezing of the samples can induce conformational changes, external validation tools are required to ensure that the vitrified samples are representative of the solution. Although many validation tools have already been developed, most of them rely on fully resolved atomic models, which prevents early screening of the cryo-TEM maps. Here, a novel and automated method for performing such a validation utilizing small-angle X-ray scattering measurements, publicly available through the new software package *AUSAXS*, is introduced and implemented. The method has been tested on both simulated and experimental data, where it was shown to work remarkably well as a validation tool. The method provides a dummy atomic model derived from the EM map which best represents the solution structure.

## Abstract 2

A novel validation tool for transmission electron-microscopy maps utilizing independent small-angle X-ray scattering measurements is introduced and implemented. The power of this technique was demonstrated by testing it using simulated data and using it on real experimental data from online repositories.

## 1. Introduction

There are three major techniques available in the toolbox for structural biology for high-resolution model determination: X-ray crystallography, nuclear magnetic resonance (NMR) and transmission electron microscopy (TEM). Until quite recently, X-ray crystallography was *the* technique for three-dimensional structure determination, which is clearly reflected in the number of biological macromolecule depositions in the online repositories (Berman *et al.*, 2000 ▸). Following a series of major breakthroughs in the field of cryogenic TEM (cryo-TEM) throughout the past decade, cryo-TEM has now become a powerful alternative to X-ray crystallography for three-dimensional structure determination. Instead of crystallizing the sample, as is required in crystallography, a series of electron micrographs for various orientations of multiple molecules are computationally merged into a single complete 3D representation (Cheng, 2018 ▸). The technique is now routinely used to obtain atomic resolution density maps of a large range of macromolecules, which can then further be used to reconstruct the complete atomic structure through a combination of model building and refinement (Pintilie & Chiu, 2021 ▸).

Small-angle X-ray scattering (SAXS) is an alternative but low-resolution technique for structural analysis. While similar in principle to X-ray crystallography based on interference of scattered X-rays, the requirement for crystallization is evaded by simultaneously measuring the scattering pattern of multiple molecules in solution. The result is a single one-dimensional orientationally averaged intensity curve that is dependent on both the shape and size of the sample. One of the primary advantages of SAXS is that macromolecular molecules and complexes can be measured in their native state in solution, without any special sample preparation. This feature is exactly what makes the technique so useful for validation.

When the Validation Task Force for three-dimensional electron microscopy convened in 2010 (Henderson *et al.*, 2012 ▸), they found a critical need for better validation methods. In response to this need, multiple Map Challenges have been held to both assess the current standard and discuss alternative avenues for evaluation metrics (Lawson & Chiu, 2018 ▸; Lawson *et al.*, 2021 ▸). Together, these meetings have resulted in many new validation metrics and methods, some of which are now an essential part of a typical map validation (Lawson *et al.*, 2020 ▸).

Since this task force was originally dispatched to incorporate validation tools into the wwPDB and EMDB depositories, they have become an integral part of any map deposition. An official validation pipeline has been deployed and directly shows useful validation metrics to the depositor. It has also become a requirement to attach such a validation report as part of the submission process for many journals (Gore *et al.*, 2017 ▸).

Validation of EM maps is primarily performed through their atomic model reconstructions. If the resolution is good enough, a complete atomic model can be built from the map itself. Typically, one would then perform both rigid-body fitting and further refinement procedures to optimize the structure. When such a model has been constructed, it can then be scored both on how well it matches the map and on how reasonable the atomic positions are through, for example, Ramachandran plots for torsion angles. Many other scoring metrics are also available; for an overview, see Pintilie & Chiu (2021 ▸), for example.

While these validation methods are all excellent for determining the quality of the map, they cannot provide information on possible tertiary and quaternary conformational changes within the sample. Such changes would typically be introduced during the sample-preparation stage during the blotting or vitrification process (Passmore & Russo, 2016 ▸). This is where our novel validation method excels. Instead of fitting an atomic resolution model to the map, it uses the independent structural information contained in SAXS measurements to validate the map. Since the SAXS data depend directly on the shape and size of the molecule, any differences between the structure represented in the EM map and in solution can easily be discovered. As the method directly uses the EM map, such a comparison does not require high-quality atomic resolution models, but can be performed using simple dummy-atom representations of the EM map. This removes one of the most time-consuming steps required for many other validation techniques, thus offering a broader range of application.

The next section will present and detail the method itself, including brief discussions of all of the major design decisions. This is followed by a section detailing how the method has been tested with both simulated and experimental data, along with tables of all test results.

## 2. Methods

In TEM, the electrons interact with the electric field generated by the individual atoms of the sample molecule. Since these fields are continuous, the surface of the molecule is not well defined in an EM map. When visualizing such a map with, for example, *PyMOL*, one must instead pick some threshold cutoff value, which is then used to define the surface. A 3D TEM map thus represent the Coulomb *charge*-density distribution, represented on a grid with a resolution dependent on the experimental setup.

In comparison, SAXS records the X-rays that are scattered by the electrons of the sample, meaning that it probes the *electron*-density distribution. While the two are similar, they are not directly related to each other. However, one can get quite far by assuming that they are proportional, as will be performed in the following. Inspired by the approach used to visualize the maps, we then introduce the idea of a variable threshold cutoff value to the method. The idea is that by varying such a threshold value, an entire series of models can be constructed from the EM map. By then calculating the expected scattering curve for each model, they can be directly compared with the measured SAXS data through the simple χ^2^ statistic. The goodness-of-fit (reduced χ^2^, 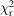) can then be used to select the best model.

**Figure 1:**
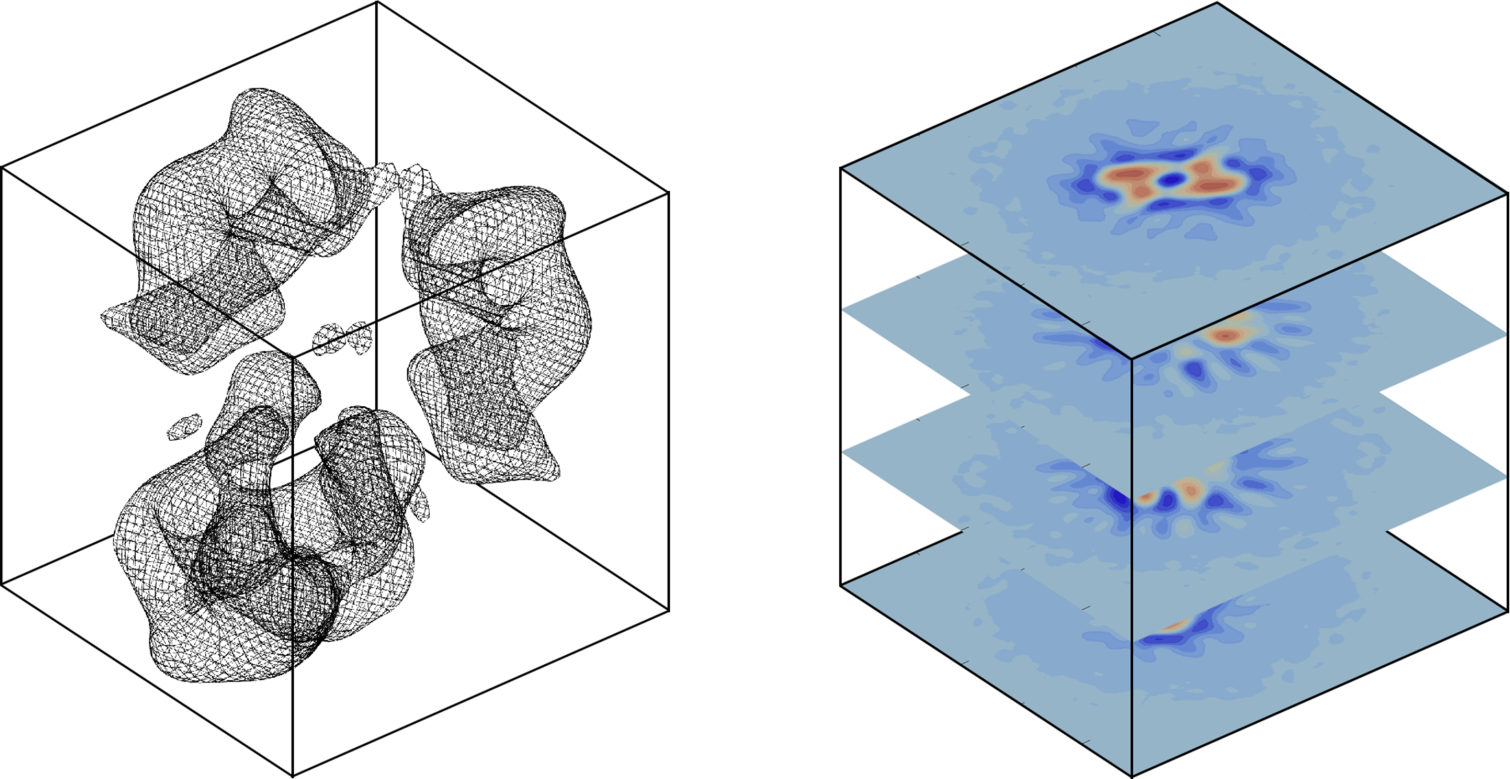
A helpful way of visualizing EM maps. The left panel shows the typical visualization from *PyMOL*. The right panel shows how the maps can also be interpreted as a stack of 2D contour plots.

Useful visualizations of a map are shown in Fig. 1 ▸. The left panel shows the usual EM map representation in *PyMOL* as a mesh grid for some given threshold (alpha level). The right panel shows an alternative way of visualizing it as a stack of 2D contour plots, with the density as the *z* axis. Varying the threshold value amounts to selecting one specific set of contour lines as a boundary. By imposing the threshold on each plot in the stack, a surface similar to the grid in the left panel is obtained.

### 2.1. Model generation

The first task is to construct a set of dummy-atom models for an EM map. The simplest way of constructing such a model is by imposing a threshold cutoff value *d*, similar to the alpha level in *PyMOL*, below which the density is assumed to be noise and is therefore discarded. The intrinsic grid of the map itself is then used to place dummy atoms, each weighted either by the density at that location or alternatively by a single default value. When using weighted densities, the grid can be seen as each coloured pixel in the contour plots in the right panel of Fig. 1 ▸. By default, each individual voxel within the threshold region is converted to a dummy atom, although depending on the map resolution every second grid point in each direction may be skipped without a significant loss of detail due to the low resolution of SAXS. It is well known that biomacromolecules in solution are surrounded by a quite well defined layer of water, which due to its ordering contributes significantly to the SAXS signal (Svergun *et al.*, 1995 ▸). We have chosen to add such a layer to the dummy-atom models, as it is expected to improve the agreement for high-resolution EM maps. These maps will typically have incomplete hydration shells due to the low scattering power of vitreous ice, hence the need to regenerate them. Thus, the final step is to simulate a hydration shell like that typically used in SAXS analyses. This is performed by randomly distributing dummy water atoms one van der Waals distance from the surface of the structure selected by the given threshold.

The creation of a single model for some threshold value thus involves first placing weighted (either by density or by some constant) dummy atoms and then simulating a hydration layer. The next step is to vary this threshold value to generate an entire series of dummy models of varying sizes. Note that the models for nearby threshold values are expected to be very similar.

### 2.2. Model selection

In the previous section, it was described how a series of dummy-atom models was generated. The next goal is to select the model that best describes the measured SAXS data. To do this, it is necessary to evaluate the expected SAXS signal of each dummy model. The simplest approach is to use the Debye equation (Debye, 1915 ▸) and then directly compare these data with the measured data through the conventional χ^2^ statistic. Selecting the best model then amounts to finding the optimal threshold value which minimizes the 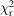.

Although there are already plenty of programs that can calculate these expected scattering curves for the models (*CRYSOL*, *FoXS*, …), we decided to use our own implementation. There are two primary reasons for this choice.

(i) As we have already discussed, we want to include a hydration layer for each model. In existing programs this is performing by adding additional terms to the form factors of surface atoms (Schneidman-Duhovny *et al.*, 2013 ▸), by adding layers of uniformly distributed electron density around the surface (Svergun *et al.*, 1995 ▸; Grudinin *et al.*, 2017 ▸), or with explicit molecular-dynamics simulation calculations (Knight & Hub, 2015 ▸). Since performing actual simulations is too slow for our purposes, we believe the best alternative is to actually model the hydration molecules as randomly distributed dummy solvent atoms close to the protein surface.
(ii) By introducing *partial histograms* into the Debye equation, a major performance improvement can be achieved when calculating the total histograms and expected scattering curves of similar structures. The idea is that by splitting the EM map into an onion-like structure with regions of similar density values, it becomes possible to reuse previous scattering calculations when scanning the threshold value. More specifically, the threshold value is scanned from its highest value to its lowest value while saving the self-correlation histogram of each ‘onion shell’. The self-correlations from the inner shells can then directly be reused when evaluating the scattering from a threshold value outside their region. Thus, instead of being a *O*(*n*^2^) process in the number of atoms, evaluating the scattering from similar structures is improved to an *O*(*nm*) process, where *m* is the number of additional scatterers. With the threshold parameter *d* being nearly continuous and by scanning from high to low, thus creating a series of similar models, *m* is small compared with *n*. Implementing optimizations such as this in existing libraries is a major undertaking, and is impossible for the closed-source *CRYSOL*. Developing a new library that natively supports these partial histograms was the easiest solution. We will return to this performance discussion later.

As an alternative to the Debye equation, one could use a multipole expansion of the scattering intensity (Stuhrmann, 1970 ▸) as performed by, for example, *CRYSOL* and *Pepsi-SAXS*, which runs in linear time, *O*(*n*). Since this approach does not use histograms and thus cannot use the partial histogram optimization, it is expected to be slower when serially evaluating the scattering signal of similar structures. Our general implementation, which can also be used for standard PDB structural models, is thus based on the Debye equation 
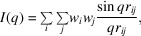
where *q* is the modulus of the scattering vector, *r*_*ij*_ is the distance between atoms *i* and *j*, and *w*_*i*_ is the weight associated with the *i*th atom. For the weights, we use *w*_*i*_ = *F*(*q*)Δρ_*i*_, where *F*(*q*) is a form factor and Δρ_*i*_ is the scattering contrast. Since we cannot distinguish between the dummy atoms created from the map, we use the simple Gaussian form *F*(*q*) = exp[−(*q*σ)^2^/2] with σ = 1 Å for all of them. The scattering contrast is set to either the map density at the location or to a common value of unity. We can extend this equation to include the hydration-shell scattering by also iterating over the dummy hydration atoms in this double sum. We assume here that the SAXS data are extrapolated to zero concentration so that no structure factor is present (Shipovskov *et al.*, 2012 ▸). We assume this to avoid introducing several additional optimization parameters into the method (Larsen *et al.*, 2020 ▸; Bærentsen *et al.*, 2023 ▸).

For highly ordered structures, such as the lattice structure of the maps, it turned out that using the binned distance approximation typically used in conjunction with the Debye equation resulted in significant inaccuracies. This is because in such highly ordered structures some distances are *much* likelier than others, yet the binning does not account for this and shifts them to the centre of the closest bin. With almost every single distance being shifted by a small amount, the error propagates into a significant uncertainty in the final scattering profile. To solve this issue, we introduced weighted bins into the approximation, where the centre of the bin is determined based on its contents, calculated as the centre of mass of the bin. This neatly solves the issue, while still providing the significant performance benefit of the binning approximation. Note that using weighted bins is usually not necessary when evaluating the scattering of a typical protein, only when dealing with highly ordered structures, as we are here.

The method optimizes four parameters in total, where the first is the threshold value itself. As explained previously, for efficiency reasons this parameter is scanned using a fixed step size, starting from its highest value and moving towards its lowest, thus generating a number of equidistant dummy models. For each of these models, three additional parameters are optimized: two for the simple linear fit to the scattering data *I*_exp_ = *aI*_model_ + *b*, and a third for fitting the scattering contrast of the hydration layer. Although adding the hydration layer generally provides a dramatic improvement to χ^2^, it also comes with a major drawback: the scattering contrast parameter is strongly correlated to the threshold value. This is only to be expected, as they both control the effective size of the model: the former by enhancing the scattering contribution from the dummy water surface atoms and the latter by directly varying the size. The strong correlation between these parameters naturally leads to large uncertainties in them, although this is not a concern as the former is an arbitrary scaling constant and the latter is only approximative. What is more problematic is the discrete nature of the data stored in the maps, with a small but finite difference between the density values of neighbouring voxels. When the threshold value crosses such a boundary, a number of new dummy atoms are added to the model proportional to the current *surface area*, while the total number of dummy atoms is of course proportional to the *volume*. Thus, for small volumes the scattering contribution of the newly added dummy atoms is significant, leading to a high variance in this region of the χ^2^ landscape. Typically, the extreme low-volume region is not of interest for the fit itself, meaning that only limited variance is observed in the relevant area of the landscape. The problem is further mitigated by using a moving average as an estimate of the actual χ^2^.

Since the threshold value is directly related to the size of the dummy model, there is in principle a one-to-one mapping from the threshold value to the total mass of the model. With this mapping the threshold axis can be replaced with a mass axis, which may be useful for real applications, especially in cases with multiple minima in the χ^2^ landscape. Since dummy models are generated for all identified minima, the user can then subsequently select only the one that they are interested in based on the mass. It should be mentioned that this mass axis comes with a significant uncertainty and may be unsuitable for absolute comparisons.

## 3. Testing

In order to test the method, a number of suitable data sets have been identified in the SASBDB (Kikhney *et al.*, 2020 ▸), EMDB (Lawson *et al.*, 2016 ▸) and PDBe (Varadi *et al.*, 2022 ▸) databases. We will henceforth refer to these data sets through their unique identifiers. As elaborated in the following, the method has been tested on both simulated and experimental data.

The ideal test would be to simulate both SAXS scattering curves and EM maps from a complete atomic structure, while also varying the resolution of the map. While the former is doable, the latter is a nontrivial problem that currently only has approximate solutions. This immediately makes this approach unusable, since one cannot determine whether a bad fit is due to issues with the map simulation or due to the method itself. We have thus focused on simulating SAXS data for our EM tests.

The methods previously implemented for evaluating the scattering from dummy structures can also be used to evaluate the expected scattering of a real atomic structure. For this purpose, the contrasts used in the equation have to be estimated. We define the contrast as Δρ_*i*_ = *Z*_eff,*i*_ − *Z*_solvent_, where *Z*_eff,*i*_ is the number of electrons, which will in general depend on the side group or compound that the atom is part of, and *Z*_solvent_ is the average electron density of the solvent multiplied by the excluded volume per atom for the molecule. For this application, the side-chain information is automatically downloaded and parsed from the RCSB PDB (Berman *et al.*, 2000 ▸) and should thus support all common side groups and compounds.

To better emulate experimental data, each point of the scattering curve should have an error associated with it. By comparison to a series of measured SAXS data sets, we have empirically found the errors to be reasonably well described by the equation 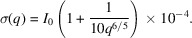
After the errors have been calculated using this equation, Gaussian noise with this magnitude is imposed on the simulated data.

### 3.1. Examples using experimental EM maps and simulated SAXS data

As part of the standard validation suites required before deposition of an EM map, a high-resolution atomic structure model is built and refined to fit the map itself. Since this fitted structure should be a good representation of the map, it can be used for testing, *i.e.* we can use the high-resolution model to generate a simulated SAXS data set for the test. We would then expect the agreement to be good, but not necessarily perfect. The tests will also serve as guidelines for the kind of results and agreements that one can expect from the method in general.

A random selection of maps covering a wide range of resolutions was downloaded for this test. SAXS measurements were then simulated for each as described above, and subsequently fitted by the scattering from the map itself as per the method described in the present paper, using unity weights.

Table 1 ▸ shows the resulting 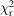 values from this test. The top table contains all of the better fits, where no obvious deviations were found between the scattering from the map and the simulated SAXS data. The 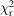 values should thus be representative of what one can expect when using high-quality EM maps without any artefacts. It is worth noting that this range of values is typical of the fits performed by other SAXS programs for high-resolution models; see, for example, Grudinin *et al.* (2017 ▸). Also shown in this table are both the expected masses and those estimated from the optimized dummy structure. We previously mentioned that the estimated masses have a significant uncertainty, which is clearly seen in the table here. Even so, the discrepancy is small enough that we believe that they may still be of use.

In contrast to this, the bottom table contains the 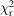 values for examples with poorer fits. These all highlight an issue or common pitfall which we would like to address.

(i) EMD-12740. The map is very porous, as if made of thousands of individual lumps. When the dummy structure is generated to calculate the scattering curve, this directly translates to a porous dummy structure, which is a poor match for the solid fitted atomic structure.
(ii) EMD-26667. The high-resolution structure used to simulate the SAXS data has a high degree of internal structure, which is not reflected in the dummy structure from the map. Together with some disagreement near the surface, a 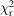 of 6 is not unreasonable.
(iii) EMD-11617. While the majority of the map matches the atomic structure extremely well, there is a small domain at the tip of the molecule which is unaccounted for in the atomic structure. This discrepancy is likely to explain the increased 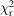.
(iv) EMD-24889. This is another case of a porous map, although it is a much worse match to its atomic structure than the EMD-12740 map was. This is likely to be due to its smaller size and lower resolution.
(v) EMD-13946. There is some disagreement between the map and the atomic structure near the flexible random coils of the protein structure, and also some minor internal disagreements. Both of these contribute to the larger than expected 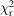.
(vi) EMD-12747. The protein is a tetramer which is open at one end, with a lower density in this region due to the disorder. Thus, when applying a threshold cutoff these disordered parts are completely left out. We will return to this map in the next section.

**Figure 2:**
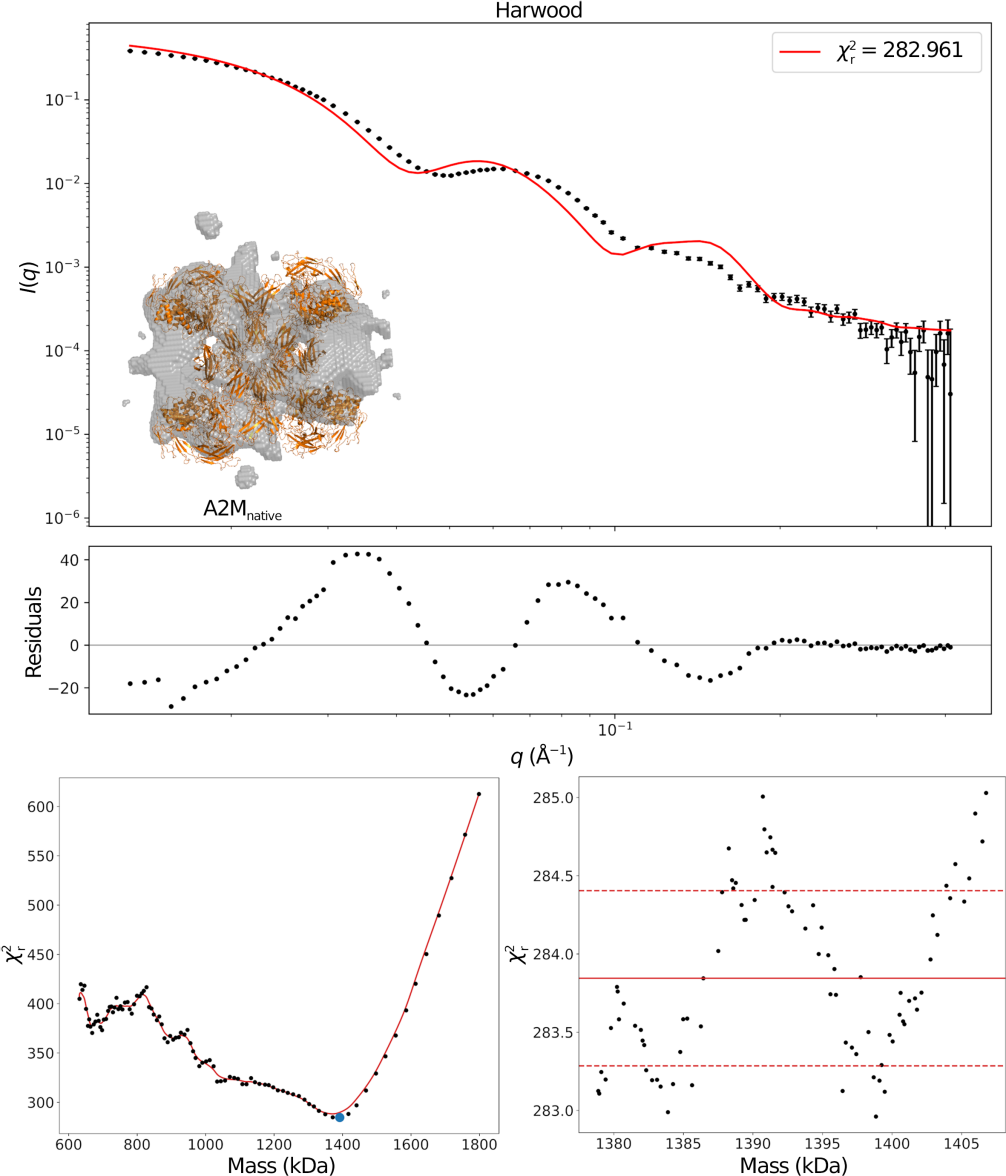
Results for native α2M using a stained EM map and experimental SAXS data from Harwood *et al.* (2021 ▸). The top panel shows the fit and the associated residuals; the inset shows the optimized dummy structure in transparent grey, with the expected atomic structure in orange. Both qualitatively and quantitatively comparing these suggests that this is a poor fit. The bottom left panel shows the χ^2^ landscape as a function of the mass, with vertical red lines indicating local minima. The right panel is an enlarged view of the area near the interpolated absolute minimum (blue dot).

Even though there were some issues with these six examples, they still only give 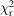 values of around 7, indicating that there is still some agreement. When the agreement is very poor (or even nonexistent), one can expect 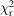 values ranging from a few hundreds to thousands. An example of this is from a negative-stained EM and SAXS study of native α_2_-macroglobulin (a2M; Harwood *et al.*, 2021 ▸). Applying our method to the data results in 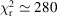 (see Table 2 ▸ and Fig. 2 ▸), indicating very poor agreement between the published map and the experimental SAXS data. Recently, a high-resolution cryo-TEM map and a corresponding atomic structure of the molecule have been published (Luque *et al.*, 2022 ▸), showing large deviations from the structure published based on the negative-stained EM map (Harwood *et al.*, 2021 ▸) discussed here.

The analyses performed here shows that one should always be aware of the quality of the map and that the conditions used for SAXS are identical to those used for EM before making comparisons with the method.

### 3.2. Examples using fully experimental data

The method has also been used on some examples where both an experimental EM map and SAXS data are available, although for the most part they were not measured by the same group. The resulting 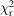 values are shown in Table 2 ▸, sorted by molar mass of the complexes. Two 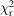 values are listed for each example. The first, 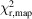, is the *absolute minimum* goodness-of-fit found during the fit of the map to the measured SAXS data, while the second, 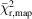, is the *average* goodness-of-fit in a small area around the minimum for the same fit. 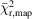 is necessary as the χ^2^ landscape may have small local variations in some cases, as discussed earlier. We also included both the expected mass *M*_W_ and the mass 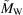 of the optimized dummy structure to evaluate the accuracy of this feature. We note that it is surprisingly difficult to find both an EM map and an associated experimental scattering curve for the same molecular complex in the literature, so the number of test cases is limited. Although obtaining the map and scattering curves from different sources is not ideal due to potential variations in the environmental conditions, these were the only examples available. Ideally, both measurements should be taken from the same sample under identical conditions. Note also that we use *all* data points from the downloaded data sets, even though they tend to be oversampled in the high-*q* region. Generally, we recommend rebinning such data to obtain a better representation of the information content at high *q*, as we have performed with our in-house SAXS data (see Fig. 4). Data sets with more realistic errors are expected to have somewhat higher 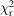 values.

**Figure 3:**
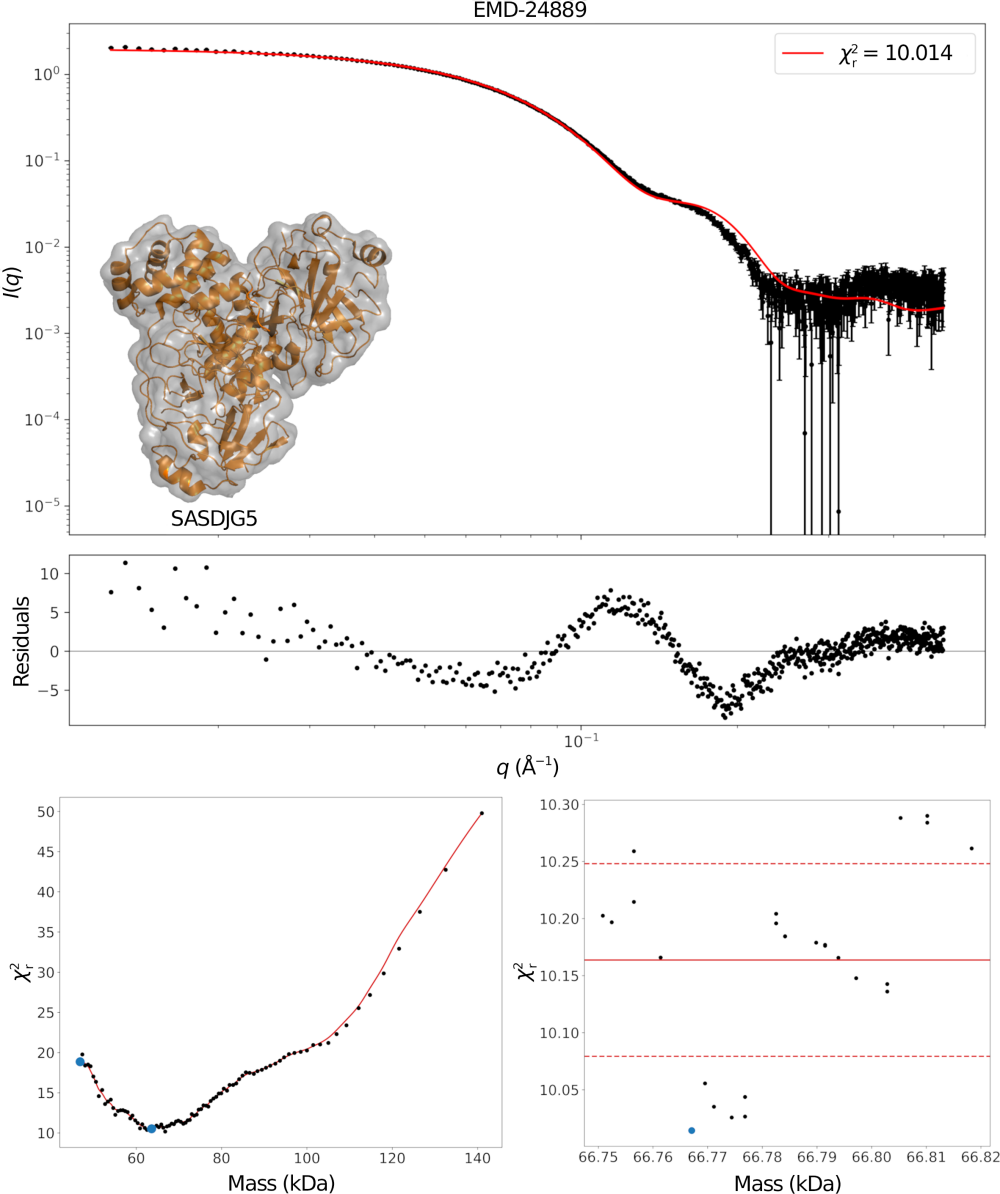
Results for SASDJG5. The top panel shows the fit and the associated residuals. The small inset shows the optimized dummy structure in transparent grey, with the atomic structure deposited alongside the SAXS data in orange. Qualitatively comparing these suggests that this is a good fit. The bottom left panel shows the χ^2^ landscape as a function of the mass, with the blue dots indicating local minima. The right panel is an enlarged view of the area near the interpolated absolute minimum (blue dot), which illustrates why an averaged 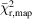 is necessary.

The fit for SASDJG5 is displayed in Fig. 3 ▸ as a double-logarithmic plot. The top panel shows the fit itself along with its residuals. Some structure is clearly visible in the residual plot, indicating that there are some issues with the model. The bottom two panels show 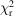 as a function of the threshold cutoff value. The left panel shows the global landscape, while the right panel shows a small region around the minimum (the blue point in the left panel). Although the minimum is well defined in this case, this will not always be the case. Thus, to avoid outliers and to improve the reproducibility of the fit, the area around the minimum is averaged to obtain 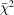, which is a more honest estimate of the minimum. Note that it is the absolute minimum 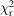 which is listed in all figures since they can only represent a single model.

Looking at 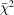, we see that the results for SASDDD3 and SASDEM9 are both in line with what can be expected when the EM map is a good representation of the solution structure as measured by SAXS. The remaining fits (with the exception of the Harwood map) all have somewhat higher 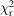 values than expected, warranting further investigation. The masses from the optimized dummy structures are generally quite far from the expected masses, even further than they were for the previous test maps, again illustrating why we stated that they should not be used for absolute comparisons. Even so, they are still useful for obtaining a rough idea of the size of the fitted dummy structure.

As we have previously mentioned, most EM map depositions also include a high-resolution atomic structure representative of the map. Although this structure is not used in our method, it is still relevant to visually compare against it, since it typically gives a good fit to the SAXS data. This visual comparison can be seen in Supplementary Figs. S1–S3, where the maps and structures have all been manually aligned, both in space and in threshold cutoff level, to give the best visual agreement. The maps for SASDEL9 and SASDEM9 could not be aligned since their resolutions were too low. These visualizations will be a great aid for the following discussion.

(i) SASDJG5 and SASDME5. All of these maps, EMD-24889, EMD-25044 and EMD-26667, are somewhat porous and have similar 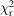 values. This porous structure means that they have a significant amount of internal structure, which is likely to cause this similar increase in the 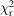.
(ii) SASDEL9. The EcTFE map is from negative-stained EM and is of low resolution. It does not appear to be a good match to the structure; in fact, the agreement is so poor that it could not even be manually aligned, thus explaining why it is not presented along with the other structures in the visualization figures. Although the SASDEM9 map, anEcTFE, is also of low resolution, it is in better agreement with the corresponding SAXS data. This is likely to be due to it being both larger and more spherical, thus reducing the resolution necessary to accurately represent it.
(iii) A2M_native_. As already mentioned, the EMD-12747 map is a tetramer with lower density at one end due to its being disordered. This means that when applying a threshold cutoff value, most of this area will be removed, thus explaining the low fitted mass. This can also be seen visually as the parts of the structure reaching out of the map in Supplementary Fig. S3. The second map, EMD-12748, suffers from the same density issue, but results in a smaller 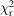. This is unexpected as it is a slightly different conformation, although with almost one half of the map missing it is not surprising that this can happen, especially when considering the even more unrealistic fitted mass.
(iv) A2M_tryp_. The high-resolution structure is a good match to the EMD-12753 map, except for the two additional internally bound trypsins that are not present in the map, one of which can be seen at the top of the leftmost panel. The map also appears to be missing some internal structure. Again, the second map, EMD-12752, is a slightly different conformation.

**Figure 4:**
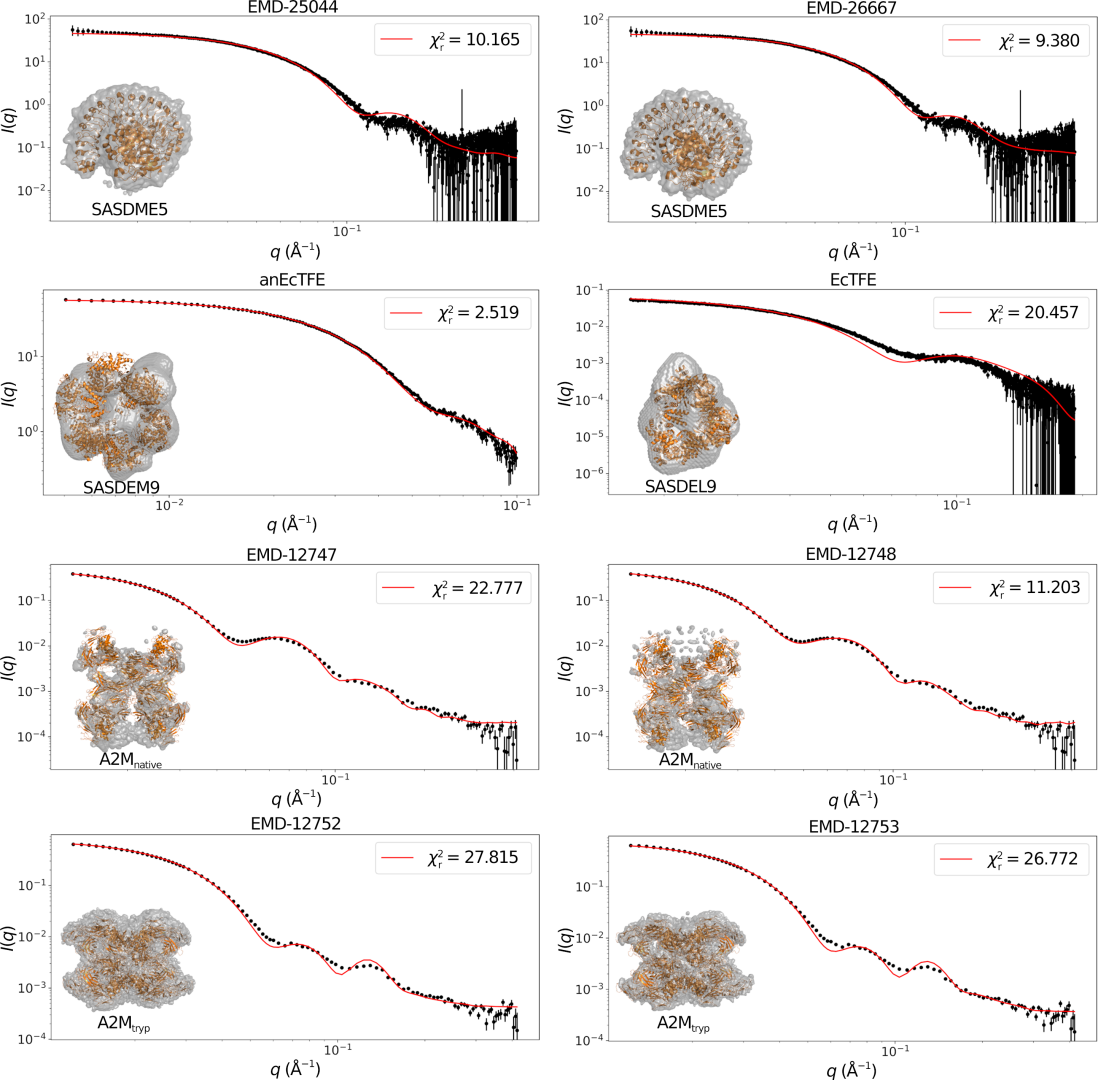
The resulting scattering profiles from using the presented method with both experimental SAXS data and matching EM maps. The small inset shows the optimized dummy structure in transparent grey, with the atomic structure deposited alongside the SAXS data in orange. Qualitatively comparing both the scattering profiles and the optimized dummy structures suggests that these are all good fits, thus successfully validating the EM maps.

Fig. 4 ▸ shows the fitted scattering profiles for most of the remaining examples from Table 2 ▸. Although the 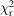 values are generally quite high, a visual comparison from this figure shows that the main features of the fitted profiles are in the correct *q*-ranges, making it a visually good fit. Here, it is once again relevant to compare against the Harwood fit in Fig. 2 ▸, where there is a clear offset in the *q*-range of the main features in the fitted profile. A second point of comparison are the optimized dummy structures, which are shown in the bottom left corner in each of these plots as a grey outline, with the atomic structure deposited alongside the fitted SAXS data shown in orange. Since the orange structures were deposited along with the SAXS data, these are expected to be in good agreement with the experimental scattering profile, and are thus relevant for comparison with the dummy structures. Again the dummy structures from Fig. 4 ▸ are in good agreement with the experimental data, while the Harwood structure is clearly in disagreement.

Based on the tests performed here, it appears that while great fits with a near-perfect 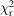 are theoretically possible with our method, in practice lower double-digit values can be expected for a typical fit with purely experimental data. Although from a purely statistical standpoint this would imply that the fits are poor, considering how the method compares data from completely different techniques and equipment this does not seem justified here, and we believe such values to be acceptable, especially in light of the profiles shown in Fig. 4 ▸.

The kind of analysis that we have performed here is exactly the intended application of the presented method. The inputs are an experimental SAXS data set and an EM map from the same molecule or complex. The program then determines the agreement between the two by using a scattering curve calculated from the map. When the agreement is good, the map has successfully been validated. When the agreement is poor, further examination of the map for spurious effects and the fitted scattering profile is warranted.

### 3.3. Alternate weighting

We mentioned earlier that two weighting modes are supported: using the densities from the map itself (dynamic weights) as the scattering weights of the dummy atoms or alternatively using a single weight of unity for everything (unity weights). Through the tests performed here, we found that using unity weights is the best option since it results in more realistic mass estimates and dummy structures. This is somewhat counterintuitive, as one would think that using all of the information contained in the map would result in more accurate calculations. The following arguments explain why this is not the case.

(i) The EM density is *per se* not the same as an excess electron-density map as probed by SAXS. Furthermore, the averaging and normalization procedures involved in the processing of EM maps may reduce the similarity to excess electron-density maps even further.
(ii) With dynamic weights, including low-density noise barely impacts the calculated scattering profile and thus the fitting algorithm is free to include it as long as it can improve the fit. Since this noise is typically not connected to the main structure but is still hydrated, including it grants the fitter more freedom to shape the profile and thus typically leads to lower 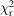 values. With unity weights the noise will always make a significant contribution to the curve and thus will be rejected by the fitter.
(iii) Since flexible parts of the protein will typically have lower density values than the more rigid parts, they will not contribute as much to the scattering profile as they should. Using unity weights solves this issue.
(iv) The last point is related to how volumes are handled internally in our method. With our grid approach, each volume cell will either be occupied or not; there is no in between. Ideally, these cells should instead contribute some percentage based on the dynamic weight of the atom occupying it, since this is proportional to how likely it is that the atom is actually found at this location. This is likely to be one of the primary reasons why dynamic weights are so prone to overestimating the total volume.

### 3.4. Benchmarking

In a field with so many alternative options for performing SAXS fitting, it is important to check and evaluate how our method compares with the alternatives. To do this, we have used the *hyperfine* program (Peter, 2023 ▸) to perform benchmarking. We tested the three programs *Pepsi-SAXS*, *FoXS* and *CRYSOL* along with our own method. Since our implementation uses multi-threading by default, a single-threaded benchmark is also provided. Based on the resource usage during the benchmarking process, along with the method description in the corresponding articles, only *Pepsi-SAXS* also seems to utilize multi-threading. The benchmarking was carried out on a Linux desktop equipped with an AMD Ryzen 5 2600X six-core processor (thus supporting a total of 12 concurrent threads with hyper-threading). The number of runs for each benchmark depends on the runtime and varies between ten and a few hundred for the smallest proteins.

**Figure 5:**
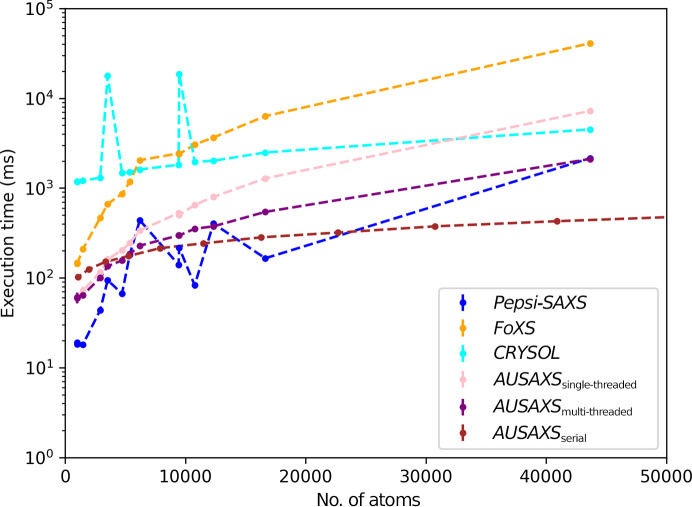
Benchmarking of the different fitting programs. *AUSAXS*_single-threaded_ is the single-threaded implementation of our method, while *AUSAXS*_multi-threaded_ is the multi-threaded implementation. *AUSAXS*_serial_ is the relevant benchmark for this paper, as it represents the average execution time for evaluating multiple similar structures around a given size. The error bars are too small to be seen on this figure. The data can also be found tabulated in Supplementary Tables S1 and S2.

Firstly, we benchmarked the fitting of a single high-resolution structure to a measured SAXS data curve. We used a selection of data sets from SASBDB with a varying number of atoms to obtain a sense of the general scaling. All H atoms and waters were stripped from the data sets, since the programs deals with these in different ways that we are not interested in measuring here. The result of this benchmark is shown in Fig. 5 ▸. Based on this benchmark, *Pepsi-SAXS* is clearly the fastest option overall, although our own multi-threaded variant becomes competitive for larger structures. In all cases both *Pepsi-SAXS* and our own implementations are significantly faster than both *FoXS* and *CRYSOL*. It is also clear that multi-threading becomes increasingly relevant for larger structures where the workload increases, which is only to be expected.

Next, we benchmarked the serial fitting of 100 similar structures to highlight the power of our partial histograms. Since this only affects the benchmark of our own implementation, the others have not been repeated. We chose to use the EMD-24889 cryo-EM map from EMDB, since it has a broad density range with a good number of voxels throughout most of it. We then selected 100 threshold values within some small range from which to generate dummy structures, and then subsequently fitted their calculated scattering curves to the experimental SAXS data. Each threshold range is then benchmarked five times to avoid outliers. The result of this benchmark is also shown in Fig. 5 ▸. With this benchmark the benefit of using partial histograms is immediately obvious: calculating a scattering curve from a large structure is *significantly* faster on average, allowing us to work with even very large systems. For smaller structures this approach is somewhat slower due to the increased bookkeeping that is required to use the partial histograms.

This benchmark is in agreement with that performed in the *Pepsi-SAXS* paper (Grudinin *et al.*, 2017 ▸), although it disagrees with the *FoXS* paper (Schneidman-Duhovny *et al.*, 2013 ▸), where *FoXS* was found to be fastest overall. It appears that only smaller structures were benchmarked in this case, for which *FoXS* is indeed faster than *CRYSOL*.

### 3.5. Comparison with other methods

Although our method is unique, the idea of combining TEM and SAXS is not. Recently, the *DENSS* (Grant, 2018 ▸) and *XMIPP* (Jiménez *et al.*, 2019 ▸) program packages have been updated with similar options. In the following, we will briefly discuss and compare against these two methods.

In a recent update (Chamberlain *et al.*, 2023 ▸), *DENSS* added the option of fitting *simulated* EM maps using SAXS measurements through the *mrc*2*sas* program. However, this new addition to the software is more directed towards verifying the primary program, which creates EM maps from atomic structure files (meaning that they are actually *electron*-density maps and not EM maps). Using it on the data presented in Table 2 ▸ results in 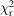 values ranging from a couple of hundred to a few thousand, except for the EMD-26667 map, where it gives a 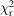 of just 23. Due to these high 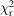 values and the fact that it was not specifically designed for this use, we do not find it relevant to perform further specific comparisons with this approach.

The *XMIPP* image-analysis suite also recently added a validation tool for TEM maps using SAXS measurements. Starting with an EM map, the method first performs a low-pass filter with a typical cutoff of 15 Å to discard any high-resolution information. A coarse-grained pseudo-atomic representation of the map is then obtained by iteratively placing Gaussian dummy atoms until the approximation error is sufficiently small. Scattering profiles from the pseudo-atomic representation are then calculated using *CRYSOL* (Svergun *et al.*, 1995 ▸). While this tool is designed to select the best-matching map from multiple options based on the scattering profile, our approach extends this functionality by objectively rejecting individual maps that do not conform to the profile. This limitation is likely to be necessary due to a combination of both the aggressive low-pass filter and the use of a very limited *q*-range for the SAXS data, using only data points below *q* ≃ 0.06 Å. This is only slightly beyond the Guinier regime, meaning that essentially maps can only be rejected based on their size.

Although the methodology is similar to ours, there are some crucial differences. Firstly, we do not have to construct approximate course-grained representations; instead, we use the intrinsic grid of the map itself to accurately represent it. Due to our highly efficient scattering calculator, we also do not have to downsample the map as heavily, thus preserving the structural information necessary for accurately estimating the scattering profile. Together, these factors allow us to compare the entire *q*-range used in a typical SAXS data set.

Although this would appear to be a perfect target for comparison, one cannot compare against the examples from Jiménez *et al.* (2019 ▸) since they define a nonstandard likelihood function using the logarithms of the data, instead of the traditional χ^2^ that we use. However, based on the previous discussion, we strongly believe our method to be the more accurate method, although perhaps also somewhat slower due to the cutoff threshold scan.

A completely different approach could be to use the *EM*2*DAM* software from the *ATSAS* package (Franke *et al.*, 2017 ▸) to obtain a dummy atomic structure from an EM map in a similar way as we do here, although only for a single threshold cutoff value at a time. One can then use *CRYSOL* to generate a hydration shell and fit it to the SAXS data. By combining these two programs, one can emulate the fit performed here, although with significantly more work and less precision.

## 4. Discussion

We have introduced a novel method for map validation using experimental SAXS data and tested it using simulated scattering curves based on high-resolution structures and experimental EM maps (Table 1 ▸). This showed that when the agreement is good, 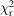 values between 1 and 4 can be achieved, in line with other SAXS fitting programs for high-resolution structures. We have also used it on examples using both experimental EM maps and SAXS data in Table 2 ▸, where it was able to validate all of the structures except for the Harwood map, as was expected. In addition to validation of the EM map, the program also provides the optimal threshold level (both in absolute value and in terms of the root-mean-square for easy plotting in *PyMOL*), along with the corresponding dummy-atom model. These are optimal in the sense that they represent the absolute minimum χ^2^ found during the fitting routine. Thresholds and dummy structures for secondary minima (if present) are also provided.

Although the method has proven to be quite useful, there are some general points that should be considered. The first is a caveat related to the different interactions of electrons and X-ray photons with matter. Since electron microscopy is based on the interaction of electrons with matter, the technique samples the Coulomb *charge* density of the molecule. In contrast to this, small-angle X-ray scattering is based on photon scattering, and thus samples the *electron* density. Although the two are somewhat similar, there are important differences. One such difference is that electrons interact with the charge of the nuclei, whereas a typical X-ray does not. One way of realizing this difference is by comparing their scattering lengths: for ionized oxygen, the electron scattering length can be negative, indicating phase shifts in the scattering process. Meanwhile, the photon scattering lengths are strictly positive since we cannot have a negative X-ray scattering length. In our approach, this difference is ignored.

Another topic is the addition of a hydration shell to the dummy structures. This is always performed by default, although this feature can be disabled. The reasoning for this is that in most cases this addition leads to a major improvement in the 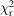 values, and in cases where it does not the fitting routine is free to set the scattering contribution from the shell to zero, which was actually utilized in some of our test cases. Although this step seems to be necessary simply based on the dramatic improvement in the 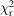 values, a physical argument can also be provided. Due to the diffuse nature of the hydration shell, its average density in cryo-EM maps is lower than in the denser parts that represent the complex. Thus, we would expect some sort of correction to the scattering power of the hydration shell to be necessary, which is typically introduced in SAXS as a free fitting parameter scaling the contribution from this shell. However, since we only have a dummy structure generated from the map, we cannot differentiate between what belongs to the protein and what is part of the hydration shell. To solve this, either some part of the outermost layer can be assumed to be the hydration shell, or an actual shell can be simulated. Since the size of the dummy structure, when varying the cutoff, is also effectively a free fitting parameter, the two approaches influence the calculated scattering in the same way. To keep the method simple and consistent, we use the latter approach: simulating the shell itself as additional dummy atoms are placed randomly on the surface. The former is also likely to run into problems with high-resolution maps with well defined borders between protein and noise.

As previously mentioned, the two fitting parameters responsible for the scattering density of the hydration shell and the threshold cutoff value are highly correlated. This is because they both control the effective size of the dummy model. If the size is increased (decreased), the scattering density of the shell can be decreased (increased) to counteract the change. Yet they are both necessary: a single parameter cannot possibly describe both effects, as the map is more structured than the simple hydration shell. This correlation, together with the noise originating from the quantized nature of the maps, mandates the use of some kind of averaging, where we have chosen to do so in an area close to the absolute minimum to obtain the goodness-of-fit values 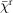.

Here, we have presented a method for the validation of EM maps. Although we have developed our own efficient implementation of the method, it is also possible to replicate some of the included procedures with existing program suites, as we have previously discussed. However, none of these existing options are able to easily and consistently replicate our method, and are mostly too impractical to be real and practical alternatives. Also, although it is possible to perform such a validation using these tools, it does not seem that the community is aware of this. Therefore, implementing all of these procedures in a single, easy-to-use program, as we have performed here, serves to make the method more known and accessible to the community as a whole.

The program is open source and freely available for academic use from its GitHub page https://github.com/AUSAXS/AUSAXS, including a graphical user interface. Comments and contributions to the implementation are welcomed there. We have also made a short user guide available in the supporting information; more detailed instructions can be found online.

## 5. Related literature

The following references are cited in the supporting information for this article: de Guzman (2019 ▸).

## Supplementary Material

Supporting information providing additional details and resources. . DOI: 10.1107/S2059798324005497/wan5003sup1.pdf

## Figures and Tables

**Table:** The listed resolution (Res.) is extracted from the map itself. *M*_W_ is the expected mass of the atomic structure as reported by the RCSB PDB (Berman *et al.*, 2000 ▸), while 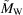 is the calculated mass of the optimized dummy structure. The upper table lists the examples with no major issues and is thus representative of the 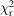 values that can be expected when the agreement is good. The examples shown in the lower table were specifically selected to highlight various issues which can cause poor fits, and were only placed in a separate table for clarity.

Map	Res. (Å)	*M*_W_ (kDa)	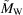 (kDa)	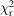
EMD-24665	1.27	498	459	1.13
EMD-27127	1.78	271	370	1.71
EMD-28025	2	660	877	3.14
EMD-15161	2.3	1404	1816	1.97
EMD-15415	2.46	245	136	3.21
EMD-25044	2.95	127	120	5.08
EMD-11616	3.06	431	412	1.70
EMD-12753	3.6	662	664	1.86
EMD-12752	4.6	661	793	4.23
EMD-12748	6.6	663	592	1.90

**Table:** 

Map	Res. (Å)	*M*_W_ (kDa)	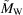 (kDa)	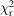
EMD-12740	1.88	121	131	6.50
EMD-26667	2.89	124	122	6.03
EMD-11617	2.94	478	531	6.11
EMD-24889	3.5	138	68	17.6
EMD-13946	3.7	146	114	6.56
EMD-12747	4.5	663	844	6.81

**Table 2:** Fit examples The listed resolution (Res.) is extracted from the map itself. *M*_W_ and 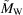 are the expected mass and the mass from the optimized dummy structure, respectively. The two A2M entries are in-house SAXS data for the native and trypsin-activated conformations from Harwood *et al.* (2021 ▸). The two TFE maps are from Sah-Teli *et al.* (2019 ▸).

	*M*_W_ (kDa)	Map	Res. (Å)	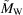 (kDa)	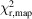	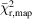
SASDJG5	68	EMD-24889	3.5	63	10.0	10.1
SASDME5	126	EMD-25044	2.95	201	10.2	10.2
		EMD-26667	2.89	385	9.4	9.4
SASDDD3	193	EMD-0560	3.2	312	2.5	2.5
SASDEL9	244	EcTFE	24	434	20.5	20.5
SASDEM9	501	anEcTFE	23	903	2.5	2.5
A2M_native_	720	EMD-12747	4.5	473	22.8	24.1
		EMD-12748	6.6	288	11.2	18.0
		Harwood	24	1398	283	284
A2M_tryp_	780	EMD-12752	4.6	1223	27.8	28.4
		EMD-12753	3.6	878	26.8	27.3
