# Supplementary material for: Validation of electron-microscopy maps using solution small-angle X-ray scattering
Source: Acta Crystallogr D Struct Biol. 2024 Jun 27;80(Pt 7):493–505. doi: 10.1107/S2059798324005497 (PMC11220840; doi:10.1107/S2059798324005497)
Supplement: Supplementary file 1 [file d-80-00493-sup1.pdf]

Volume 80 (2024)

Supporting information for article:

Validation of electron-microscopy maps using solution small-angle X-ray scattering

Kristian Lytje and Jan Skov Pedersen

This is the supplementary information providing additional details and resources to support the main article. Included is a small user guide for the operation of the distributed executable employing the EM validation method. Visualization plots supplementing the discussion of both the simulated and experimental tests are also presented. Tabulated data backing the benchmark figure in the main article is also available here.

Table S1

Benchmark times. The subscripts 'st' and 'mt' are short for 'single-threaded' and 'multi-threaded', respectively. Each value represents the mean and standard deviation of between 10 and a few hundred runs in milliseconds, depending on the runtime of each program. A (†) indicates a SASBDB id.

Data	Atoms	Pepsi-SAXS	$AUSAXS_{mt}$	$AUSAXS_{st}$	FoXS	CRYSOL
DPT4†	1001	18(1)	60(8)	61(9)	147(11)	1175(18)
DPP4†	1004	19(1)	61(8)	60(6)	144(11)	1478(74)
DPS4†	1481	18(1)	64(4)	72(8)	209(11)	1207(13)
DE35†	2917	44(2)	100(7)	116(6)	464(18)	1302(18)
DQ59†	3540	94(5)	135(10)	161(7)	665(17)	18(1)s
DJG5†	4734	67(2)	156(10)	204(10)	863(20)	1478(74)
DME4†	5380	181(11)	184(12)	244(9)	1170(32)	1511(32)
DPB9†	6210	438(21)	228(5)	336(12)	2043(54)	1609(24)
urateox	9436	140(9)	297(5)	527(16)	2426(71)	1816(36)
DPQ4†	9488	216(11)	297(6)	502(7)	2390(73)	19(3)s
DDD3†	10775	83(7)	352(7)	648(11)	3051(44)	1957(83)
DPR4†	12332	402(18)	374(10)	797(9)	3653(78)	2012(30)
DEL9†	16640	165(14)	542(9)	1279(24)	6315(174)	2488(76)
$A2M_{native}$	43652	2149(90)	2108(11)	7231(11)	41(2)s	4497(52)

Table S2

Benchmark of serial evaluation of the scattering curves of the 24889 cryo-EM map. One hundred SAXS curves are calculated within each threshold range five times. This table can be compared with the benchmarks for single structures in table S1.

Range [σ]	Atoms	Average time [ms]
[2, 3]	[192155, 148408]	1512(3)
[3, 4]	[148040, 118570]	1046(2)
[4, 5]	[118310, 95332]	828(1)
[5, 6]	[95106, 76358]	681(7)
[6, 7]	[76182, 60166]	573(1)
[7, 8]	[60006, 46584]	491(1)
[8, 9]	[46460, 35298]	427(1)
[9, 10]	[35198, 26322]	375(1)
[10, 11]	[26245, 19144]	319(1)
[11, 12]	[19072, 13542]	282(1)
[12, 13]	[13502, 9486]	242(2)
[13, 14]	[9446, 6368]	214(6)
[14, 15]	[6338, 4182]	175(1)
[15, 16]	[4162, 2558]	151(1)
[16, 17]	[2542, 1440]	125(1)
[17, 18]	[1428, 736]	102(1)

User guide to the software

We here present a short example illustrating how the program can be used. We will fit the `EMD25044` map to the `SASDME5` SAXS dataset. There are two supported modes of operation: directly using the terminal, and using the simple graphical user interface. Both executables are freely available on the project GitHub page `https://github.com/AUSAXS/AUSAXS`.

Using the terminal

From a terminal, the program can be launched through the `em_fit` executable. The basic syntax is `em_fit <map file> <SAXS file>`, where a number of options can be appended. For a full list, use the `em_fit -h` option. A more complete description of each option and what it controls is available in the online documentation.

After entering the path to the map, the SAXS file, and any required options, hit enter to start the fit. After a short setup time, the fit itself will begin. During this phase, the current threshold cutoff value and its associated χ^2 is shown in the terminal and updated for every iteration. Since the scan starts with the smaller dummy structures and slowly works its way towards larger dummy structures, the iteration rate will steadily slow down throughout this phase. After having scanned the majority of the threshold range with the requested number of steps, the fitting is allowed to terminate, after which it will enter the second phase: estimating the uncertainty of the minima. In this phase it will focus on the area surrounding the lowest minima it encountered in the first phase. This is usually quite fast, but may add significant computing time depending on the size of the dummy structure at the minima. At the end of this phase, the absolute minima is printed to the terminal along with some additional information about the optimized structure. A number of `.plot` files will also be saved to the output directory. To convert these files to actual plots, first navigate to the folder, and then run the `plot.py` Python script without any arguments.

Using the graphical user interface

The interface, built with the Elements(de Guzman, 2019) C++ package, can be started by executing (double-clicking) the `em_fit_gui` executable. This will load up a window similar to figure 2a. Here the most important options can be changed, though the more advanced ones are only available through the terminal interface. Start by hitting either of the folder icons next to the `map path` and `saxs path` fields. Navigate to the data file and open it. Doing this with the `map path` will automatically pull in the SAXS data if a file with the correct extension is also present in the same folder.

After both datasets have been loaded in, the output path will automatically change to a unique path for this combination of files, preventing accidental overwrites, see figure 2b. The output path can also be manually changed if necessary. Now the remaining options can be changed if required, though the defaults are typically sufficient. When you are ready, hit the central `start` button to initiate the fitting, after which a progress bar will be shown as seen on figure 2c. When the fit is complete, the resulting plots will be shown directly in the interface, like in figure 2d. You can now cycle through them using the small menu.

Understanding the output

There are two main plot types: the fit to the scattering profile, and the χ_r^2 landscape plots. The former comes with both linear and logarithmic *x*-axis variants, while the latter has five different plots with various zoom levels and *x*-axes.

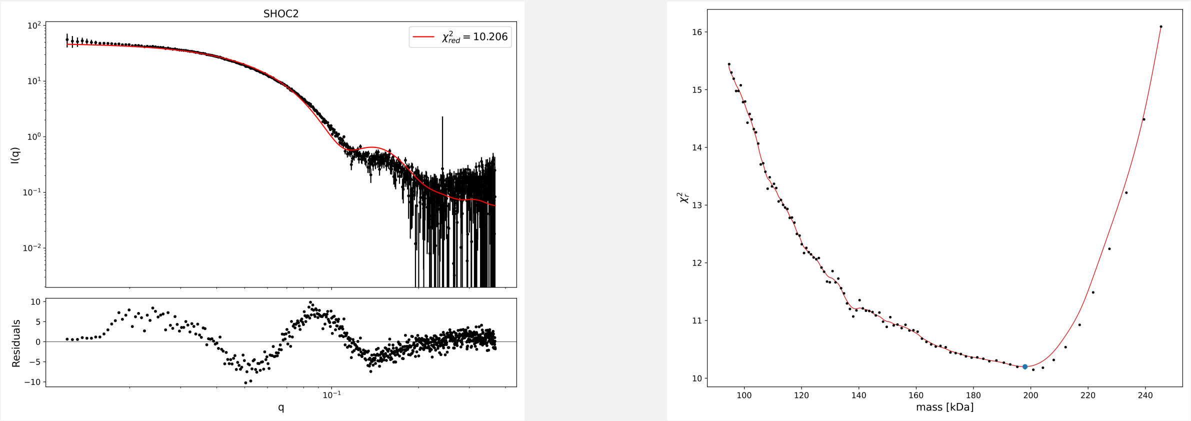

Figure 1
(*left*): The scattering profile with double-logarithmic axes. The upper half shows the data points with error bars in black, with the red curve denoting the best fit. The legend shows the goodness-of-fit through the χ_r^2 value.
(*right*): The χ_r^2 landscape with an intermediate zoom level and with the mass as the *x*-axis. The χ_r^2 landscape shown with an intermediate zoom level and with a mass *x-axis*. When analyzing the output, it is important to check if the minima is well-defined in this type of plot. Sometimes multiple minima may be present, in which case the folder `models` will be created, containing fit information and dummy structures for each such minima.

User guide to the software

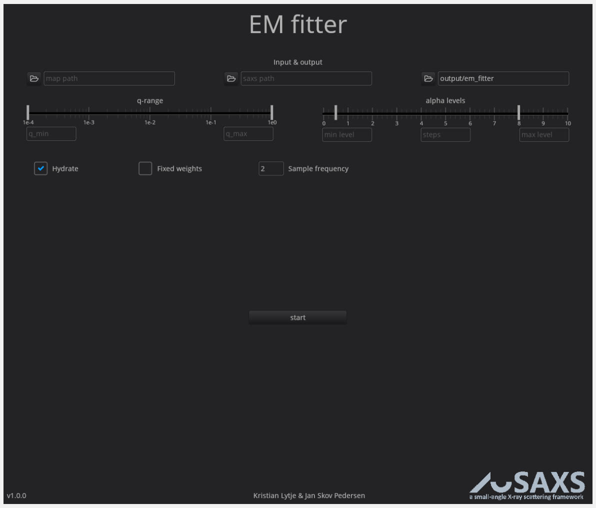

a) The interface after starting.

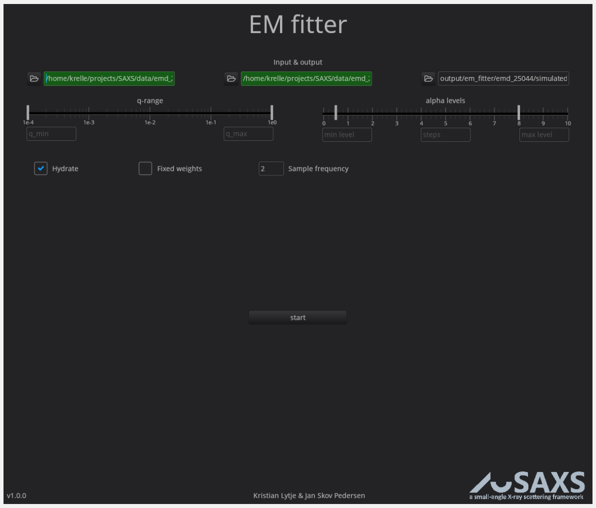

b) The interface after inputting the data. Note the green coloring indicating that the files were successfully opened.

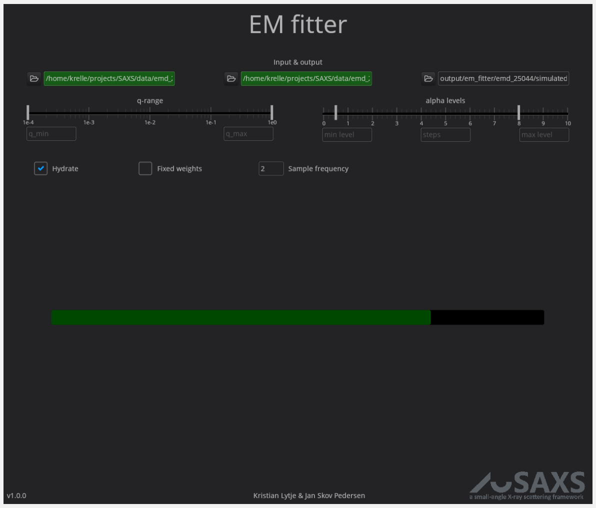

c) The interface while running the fit itself. Note that the progress bar will likely not progress linearly.

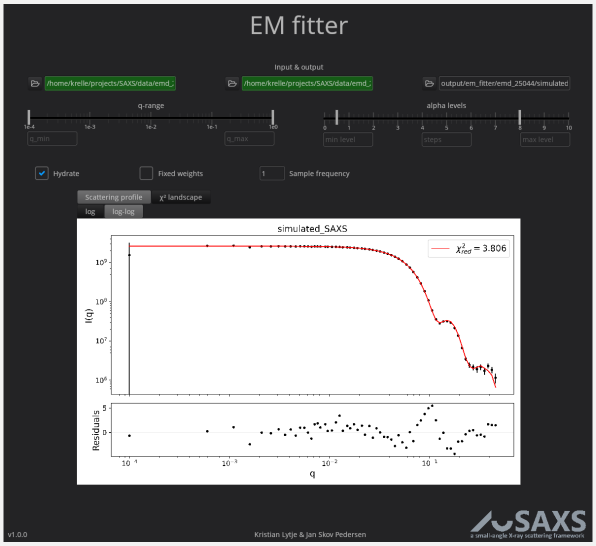

d) The interface after completing the fit. All generated figures can be viewed directly inside the program itself.

Figure 2
Example use of the graphical user interface. Note that this is an early version and may thus be subject to change in the future.

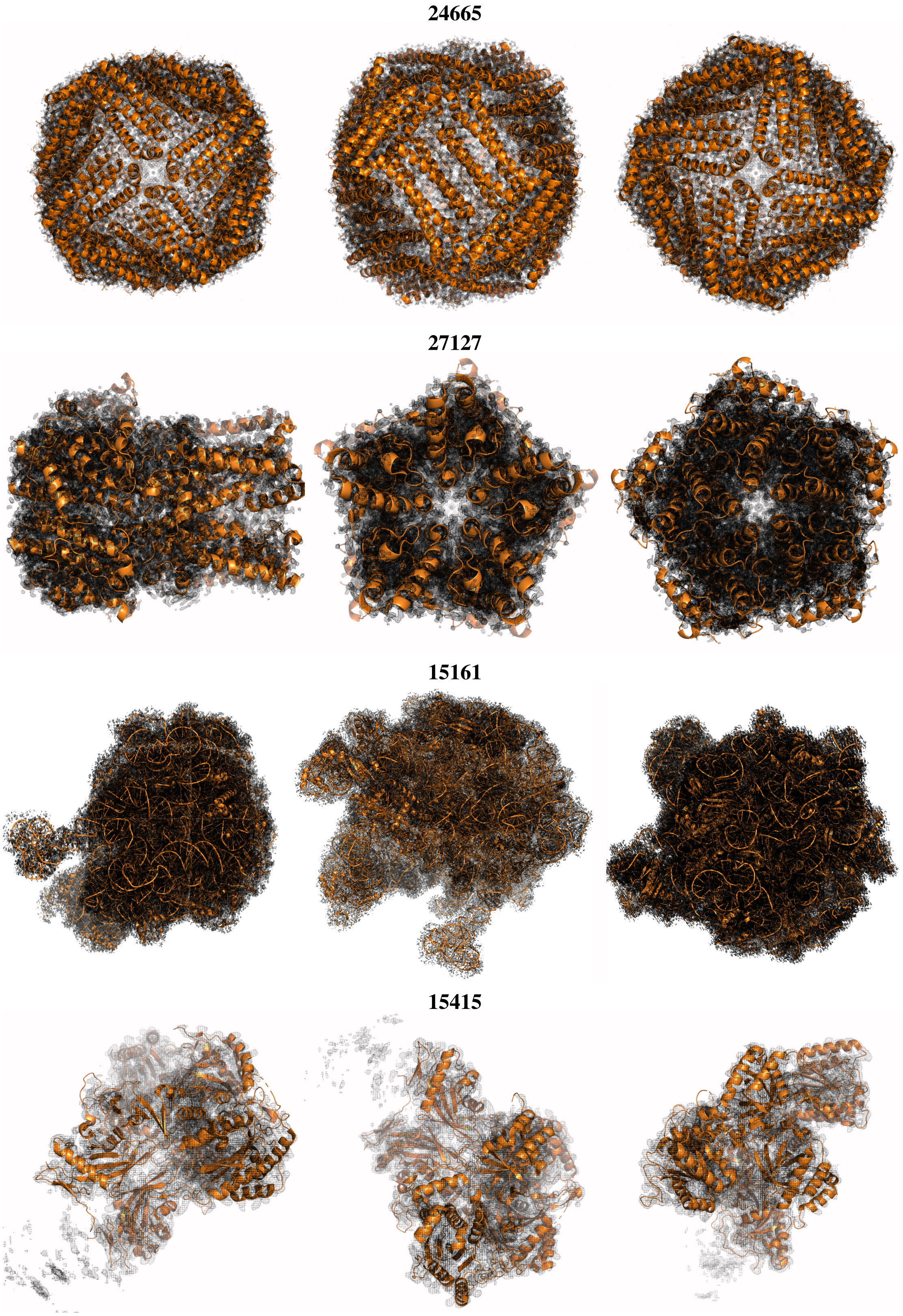

Figure S1
Projections of the test maps and an high-resolution atomic structure, showing the map and its associated structure manually aligned both in space and with the threshold cutoff value.

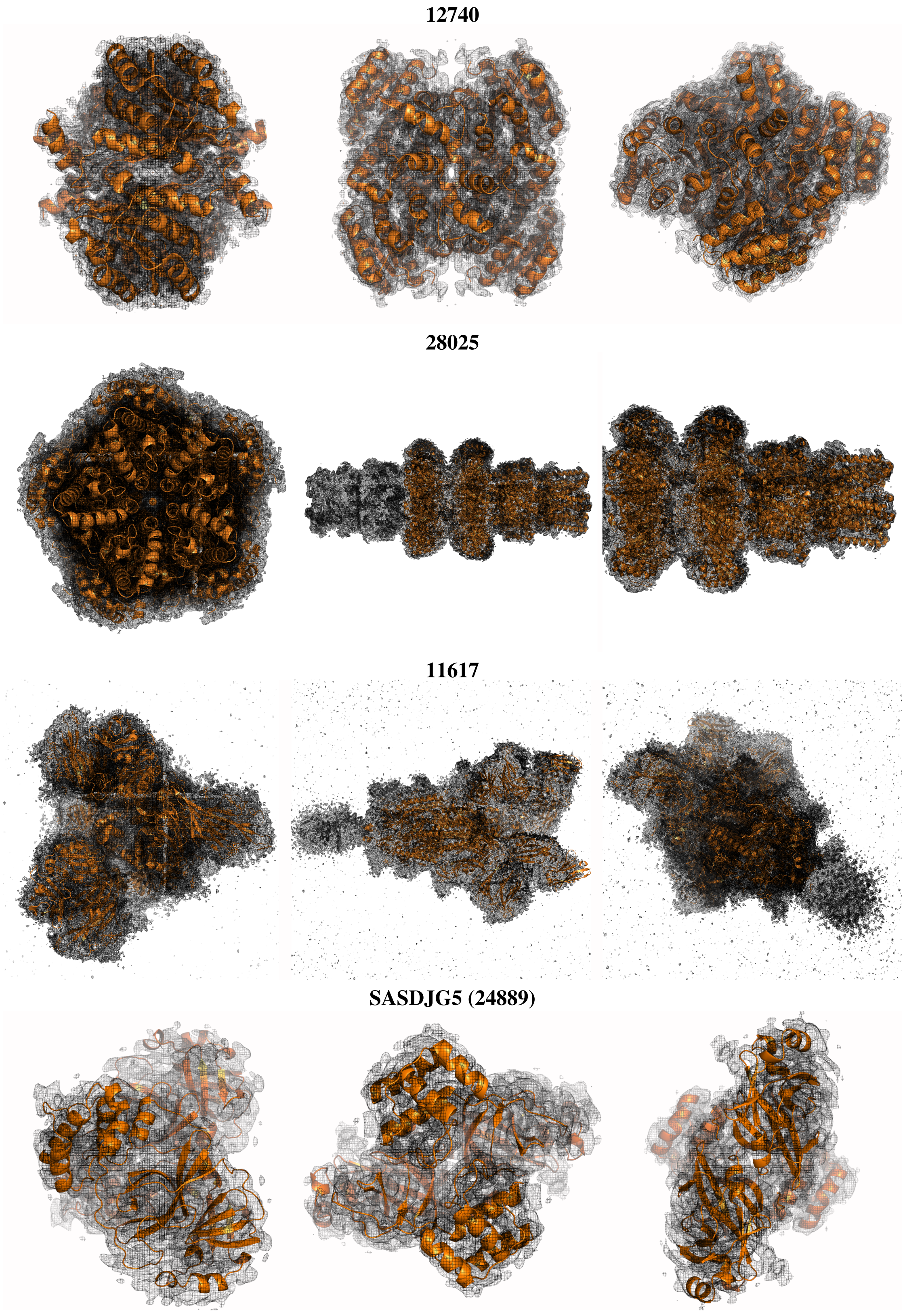

Figure S2
Projections of the test maps and an high-resolution atomic structure. For the first three proteins (denoted with an EMDB id), the structure associated with the EM map is shown. In the last, the structure associated with the experimental SAXS data is shown instead. In all cases they were manually aligned, both in space and with the threshold cutoff value.

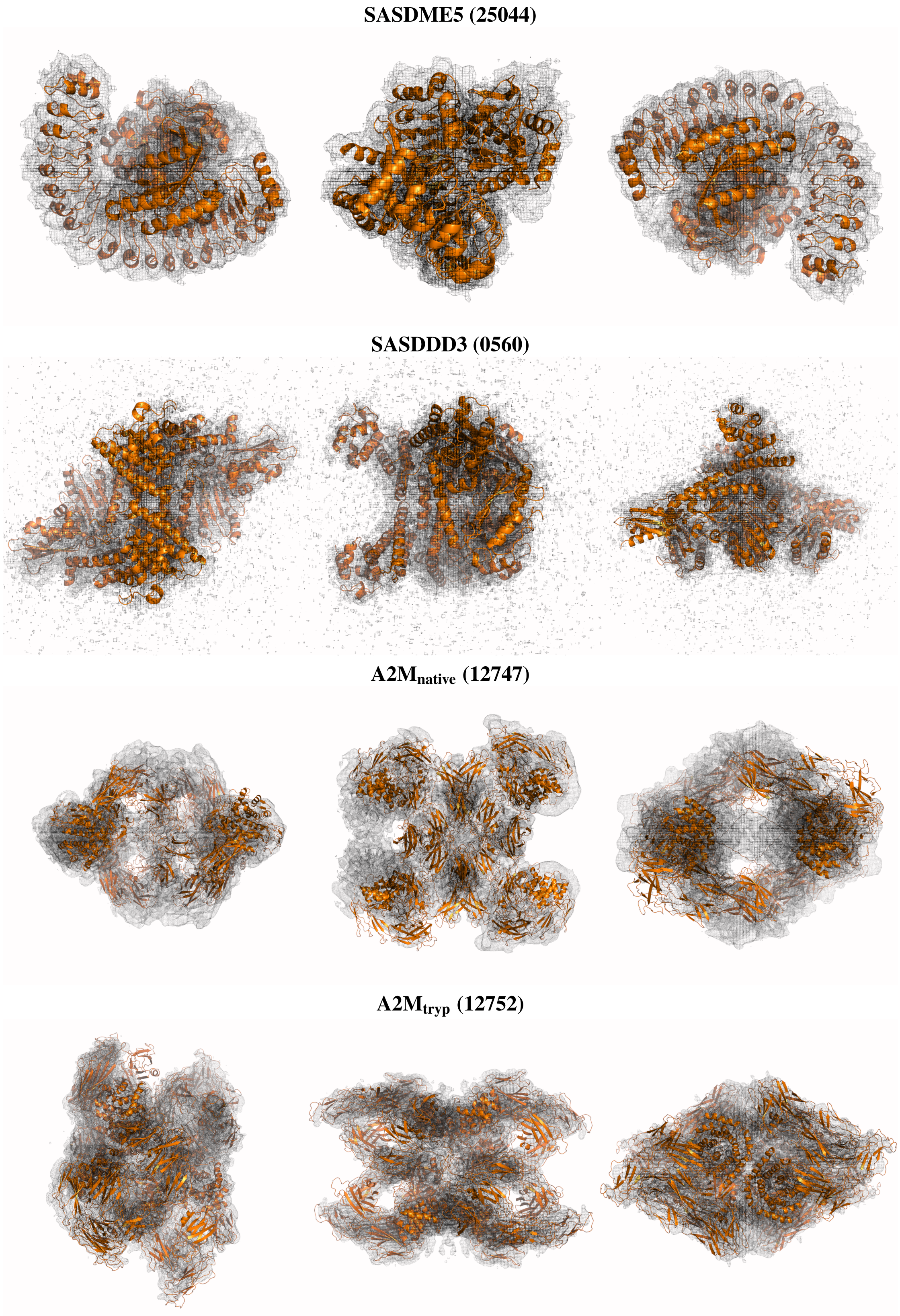

Figure S3
Projections of the test maps and an high-resolution atomic structure. For the first seven proteins (denoted with an EMDB id) the structure associated with the EM map is shown, while for the remaining five (EMDB id in parentheses) the structure associated with experimental SAXS data of the same structure is shown. In all cases they were manually aligned, both in space and with the threshold cutoff value.

References

de Guzman, J., (2019). Elements.
https://github.com/cycfi/elements
